# Myofibroblasts and increased angiogenesis contribute to periapical cystic injury containment and repair

**DOI:** 10.4317/medoral.23605

**Published:** 2020-05-10

**Authors:** Camila Tatyanne Santos de-Freitas, Glória Maria de-França, Manuel Antonio Gordón-Núñez, Pedro Paulo de Andrade Santos, Kênio Costa de-Lima, Hébel Cavalcanti Galvão

**Affiliations:** 1 Postgraduate Program in Oral Pathology, Department of Dentistry, Rio Grande do Norte Federal University -UFRN; 2 Postgraduate Program in Dentistry, Department of Dentistry, Paraíba State University-UEPB; 3 Postgraduate Program in Structural and Functional Biology, Biosciences Center, Rio Grande do Norte Federal University -UFRN; 4 Postgraduate Program in Health Sciences, Department of Dentistry, Rio Grande do Norte Federal University -UFRN

## Abstract

**Background:**

Myofibroblasts (MF) and angiogenesis are important factors in the development and expansion of cystic lesions, where these cells secrete growth factors and proteases, stimulating angiogenesis, matrix deposition and cell migration, affecting the growth of these periapicopathies. The present study aimed to evaluate the immunohistochemical expression of CD34 and α-SMA in radicular cysts (RC) and residual radicular cysts (RRC), with the purpose of contributing to a better understanding of the expansion and progression of these periapical lesions.

**Material and Methods:**

The present study os a descriptive, quantitative and comparative analysis of positive CD34 and α-SMA immunohistochemical expressions in 30 RC and 30 RRC specimens. α-SMA expression was evaluated in the fibrous capsule of the lesions, at 100x magnification below the epithelial lining. A total of 10 higher immunostaining fields were selected and subsequently, positive cells were quantified at 400x magnification, averaged per field. Regarding the angiogenic index, immuno-labeled microvessel counts for the anti-CD34 antibody were performed in 10 fields at 200x magnification.

**Results:**

Statistically significant differences regarding α-SMA immunostaining were observed (*p* = 0.035), as well as a correlation between α-SMA versus CD34 (*p* = 0.004) in RRC. However, the angiogenic index obtained by immunostaining for CD34 indicated no statistical difference between lesions. Intense inflammatory infiltrates were predominant in RC, while mild and moderate degrees were more commonly observed in RRC (*p* <0.001). Intense inflammatory infiltrates were also more often noted in larger RRC (*p* = 0.041). Inflammatory infiltrates showed no significant correlation with α-SMA and CD34 immunostaining.

**Conclusions:**

The results indicate that the significant correlation found between the presence of MF and the angiogenic index are related to the repair process in RRC.

** Key words:**Myofibroblasts, angiogenesis, inflammatory odontogenic cysts.

## Introduction

Periapical lesions are inflammatory conditions of the periradicular tissues resulting from direct sequelae of pulp necrosis infections and consequent progression to the apical region (1), linked to the fragility of host defense mechanisms in removing contamination by persistence of antigenic agents (2). Inflammation of the necrotic tooth pulp results in proliferation of Malassez epithelial remains, which are Hertwig's root epithelial sheath remnants that become stimulated to proliferate, giving rise to radicular cysts (RC) (3).

Myofibroblasts (MF) are connective tissue cells that exhibit a hybrid phenotype, morphologically presenting as large and spindle-shaped cells, exhibiting characteristics between those of fibroblasts and smooth muscle cells (4). The presentation of such a phenotype is due to a transdifferentiation process, characterized by the expression of smooth muscle α-actin (α-SMA), a protein essential for MF identification, which are recognized in cystic and tumoral lesions, controlling phenomena such as tissue reorganization capacity (4) and angiogenesis through the secretion of inflammatory mediators, chemokines and platelet-derived growth factor (PDGF) (5).

Regarding inflammatory odontogenic lesions, scarce research analyzing the expression of α-SMA and CD34 in periapical lesions is available. In this context, this study aims to evaluate the pathogenesis and growth of periapical cystic lesions assessed through immunohistochemical expression analyses.

## Material and Methods

- Study design and tissue samples

The research is a cross-sectional and retrospective study. The sample was selected non-probabilistically and for convenience based on consecutive cases and specimens derived from total surgical removal. The calculation for the sample size was determined by the prevalence of the lesions. The prevalence of RCs was 29.2% obtained by Becconsall-Ryan *et al*. (6). For RRCs, the prevalence of 4.9% was obtained by Souza *et al*. (7), adopting 5% alpha error, 80% test power and 20% beta error, 3% confidence interval amplitude, resulted in a minimum size of 28 cases for RCs and 6 cases for RRCs, according to the formula: n= (z2 pq)/(FE2).

Thirty radicular cyst (RC) and thirty residual radicular cysts (RRC) cases were obtained from the pathology records of the Postgraduate Program in Oral Pathology belonging to the Rio Grande do Norte Federal University, in Brazil between years range from January 2008 to January 2019. Only specimens presenting sufficient biological quality and quantity were selected for the study. For both cases (RC and RRC), only fragments exhibiting pathological cavity coated entirely or partially by epithelium and a sufficient amount of fibrous capsule to perform immunohistochemical evaluations were selected. Information regarding gender, age, anatomical location, symptomatology and lesion size were collected. Clinical records with insufficient information concerning these variables were excluded from the study.

- Morphological analysis

The selected cases RC and RRC cases were cut into 5 µm-thick samples, stained by Hematoxylin/Eosin (HE) technique and analyzed under light microscopy (Olympus BX41, Olympus Japan Co., Tokyo, Japan) at 40x, 100x, and 400x magnifications. Inflammatory infiltrate intensities were analyzed at 400x magnification according to an adaptation of the França *et al*. criteria (8). Nine microscopic fields were selected, divided into sets of three consecutive fields starting the epithelium analysis and extending deep into the capsule. Specimens whose inflammatory infiltrate was restricted to 1/3 of the microscopic field were considered as Grade I (mild infiltrate); lesions with inflammatory cells present in up to 2/3 of the microscopic field were defined as Grade II (moderate infiltrate); and lesions that exhibited inflammatory infiltrate in over 2/3 of the microscopic field were categorized as Grade III (intense infiltrate). At the end, the average of each set of three fields was calculated and the intensity of the inflammatory infiltrate of the case was established. The thickness of the epithelial lining of the cysts was analyzed according to the methodology proposed by França *et al*. (8). Under this perspective, cystic epithelia presenting from 2 to 10 layers in their greatest extent and hyperplastic above 10 layers were considered atrophic.

Data related to the size of cystic lesions were obtained by macroscopic measurement and were categorized into three groups according to their largest dimension in centimeters (cm): group 1 (≤ 2 cm); group 2 (> 2 to 4 cm) and group 3 (> 4 cm) (9).

- Immunohistochemical analysis

The α-SMA immunostaining (DAKO, Carpinteria, CA, USA), clone (1A4), specification (Monoclonal), dilution (1:500), antigen retrieval (Trilogy), incubation (60’) was performed to identify myofibroblasts. The CD34 antibody (CELL MARQUE, MilliporeSigma, USA), clone (QBEnd/10), specification (Monoclonal), dilution (1:200), antigen retrieval (Trilogy), incubation (60’) was used to determine the angiogenic index.

Histological 3 µm thick sections were obtained and spread on previously cleaned glass slides prepared with 3-aminopropyltriethoxy silane adhesive (Sigma Chemical CO, St Louis, MO, USA). The material was subsequently subjected to the antibodies through the immunohistochemistry technique applying the streptoavidin-biotin method (LSAB-estroptoavidin biotin complex).

Positive controls for the α-SMA and CD34 antibodies were performed with histological capillary hemangioma sections. The negative control consisted of replacing the primary antibody with 1% bovine serum albumin (BSA) in a buffer solution, followed by all subsequent steps of the protocol described as follows: 1) All samples were immersed in a prepared solution containing Trilogy® in one contained, followed by tissue sample immersion in a paschal pot for deparaffinization, rehydration and antigenic site recovery; 2) The material was then washed under running water for 3 minutes with two distilled water washed for 1 minute each; 3) The samples were then incubated for 15-minutes in a 10-volume hydrogen peroxide solution (3%) to block tissue endogenous peroxidase; 4) All samples were then washed with distilled water; 5) Samples were then immersed in a 1% Tween20 solution in TRIS-HCl pH 7.4 for 5 minutes each; 6) Samples were then incubated with Background Block protein (Cell Marque, Rocklin, California, USA) for 10 minutes; 7) Samples were incubated with diluted primary antibodies (Envision Flex Antibody Diluent - DM830), for 60 minutes; 8) Secondary antibody incubation was then carried out using the Hi Def Detection System (Cell Marque; Rocklin, California, USA) applying the amplification solution for 20 minutes; 9) The HiP System HRP polymer was then applied for 20 minutes; 10) Application of the DAB chromogen agent (diaminobenzidine; Scytek Laboratories; Logan, Utah, USA) was then performed for 5 minutes at room temperature; 11) Samples were then counterstained with Mayer's hematoxylin at room temperature for 5 minutes; 12) quick washes in distilled water (three changes) were then performed; 13) Ethanol upstream dehydration was performed, as follows: Ethyl alcohol 80° GL (3 minutes), Ethyl alcohol 95° GL (3 minutes), Absolute Ethyl Alcohol I (5 minutes), Absolute Ethyl Alcohol II (5 minutes), Absolute Ethyl Alcohol III (5 minutes), Xylol I immersion (5 minutes) and, finally, Xylol II immersion (5 minutes).

- Immunohistochemical profile analysis

Each case was analyzed by 2 pre-calibrated examiners under light microscopy (Olympus BX41, Olympus Japan Co., Tokyo, Japan). The slides were then scanned (Panoramic MIDI, 1.15 SPI, 3D HISTECH, Budapest, Hungary) and imaged using the Panoramic Viewer 1.15.2 software (3D HISTECH, Budapest, Hungary).

MF identification was performed by α-SMA immunostaining based on the methodology proposed by Vered *et al*. (10). A total of 10 subepithelial fields with higher immunoreactivity were selected using a 100x magnification. Positive cells were quantified in each of these fields at 400x magnification, eliminating those in the periphery of blood vessels. The obtained values were summed and subsequently the positive cells were averaged per field and for each case, individually. The examiners analyzed the lesions separately and any disagreements were reassessed until a consensus was reached.

The angiogenic index was obtained through microvascular counting (MVC), using an adaptation of the Maeda *et al*. method (11). Thus, 10 fields located below the epithelial component with the highest immunostaining for the CD34 antibody were selected at a 100x magnification. Subsequently, microvessels were quantified in each of these fields at 200x magnification. The obtained values were then summed, establishing the total number of microvessels. Finally, the average number of microvessels per field was calculated in each case.

- Statistical analyses

The immunohistochemical analysis results were entered into an Excel spreadsheet (Microsoft Office 2016®) and later exported to the Statistical Package for the Social Sciences program (version 24.0; SPSS Inc., Chicago, IL, USA), via the freeware version, where the statistical analyses were performed.

After applying the Shapiro-Wilk test to determine data normality, the statistical analyses were performed using the nonparametric Mann-Whitney test to verify statistical differences between lesion inflammatory infiltrate degree and α-SMA and CD34 immunostaining between lesions.

Spearman's correlation test was used to cross quantitative variables, such as α-SMA immunoexpression, CD34 and lesion size in cm, in addition to the ordinal grading of the inflammatory infiltrate. Finally, Pearson's chi-square association test was used to analyze the association between epithelial thickness between RC and RRC. The confidence interval for all analyses was set at 95% and *p* <0.05.

## Results

- Epidemiological and Clinical Results

The sample of the present study was intentional and consisted of 60 cases, 30 RC and 30 RRC. Regarding gender, female samples were predominantly in RC (4:1 F:M; n=24; 80.0%) while male samples were predominantly in RRC (2:1 F:M n=20; 66.7%). Regarding anatomical location, the anterior region of the maxilla was the most affected site for both lesions (n=16; 53.3%), followed by posterior region of mandible (n=6 in RCs; n=6 in RRCs), posterior region of maxilla (n=6 in RCs; n=5 in RRCs) and anterior region of mandible (n=2 in RCs; n=3 in RRCs). The mean age for RC cases was 34.1 ± 13.21, while the mean age for RRC cases was 52.8 ± 13.34 years. Regarding symptoms, asymptomatic cases were predominant for both RCs (n=22; 73.3%) and RRCs (n=21; 70.0%).

- Morphological Results

Regarding epithelial thickness, a significant association was observed between the epithelial lining and type of lesion (Table 1). RC displayed a higher frequency of hyperplastic epithelium compared to RRC exhibiting an atrophic epithelium (*p* = 0.037).

The analysis concerning inflammatory infiltrate intensity revealed the predominance of an intense inflammatory infiltrate degree in RC, while higher mild and moderate degree frequencies were observed for RRC (Table 1).

Regarding lesion size, RRC were larger compared to RC, presenting an average size of 2.21 ± 0.92 cm in diameter compared to 1.90 ± 1.63 cm in diameter, respectively, considering macroscopic measurements. The RC median was of 1.4 (0.5-2.2), while the RRC median was of 2.0 (1.4-3.0).

RC did not present significant correlations between size and inflammatory infiltrate, while a significant positive moderate correlation was found for RRC cases (*p* = 0.041) (Table 2).

**Table 1:**
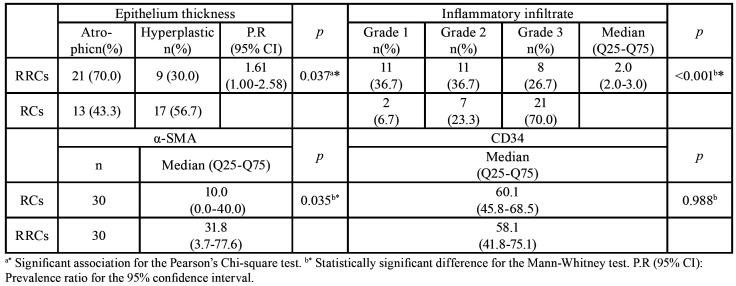
Morphological and immunohistochemical characteristics of periapical lesions.

**Table 2:**
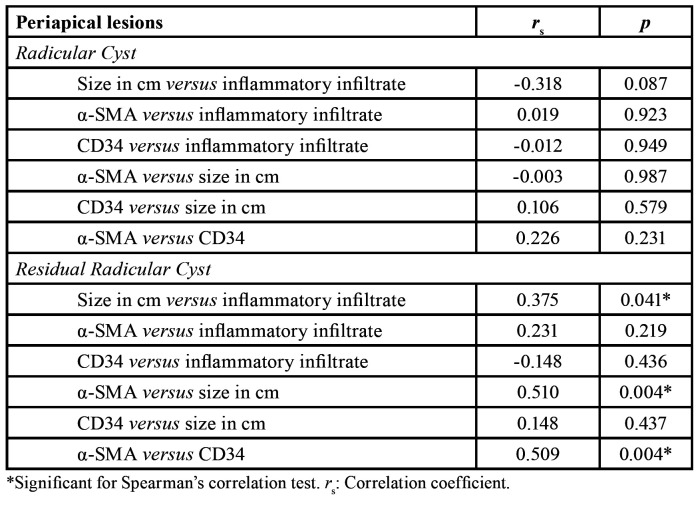
Correlation between α-SMA and CD34 immunoexpression in inflammatory infiltrate and size of cystic lesions.

- Immunohistochemical Results

α-SMA immunoexpression

**Figure 1:**
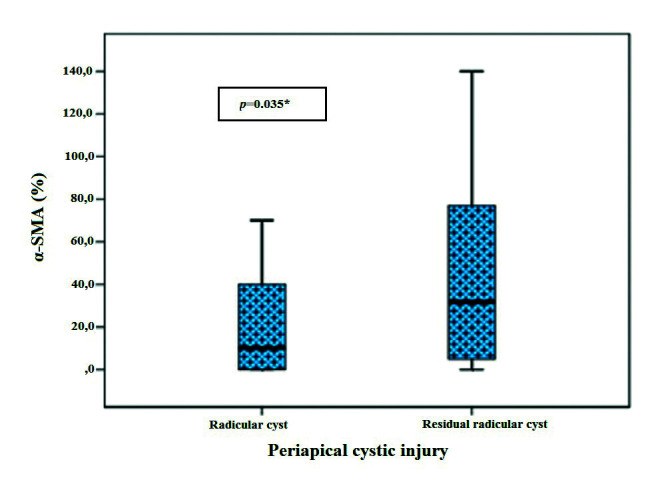
α-SMA immunostaining box-Plot in RC and RRC.

MF were identified by immunostaining for α-SMA antibody in most analyzed cases. The average for RC cases was of 23 MF per field and a median of 10.0 (0.0-40.0). For RRC, average MF consisted in 42 cells per field and a median of 31.8 (3.7-77.6) (Table 1). A statistically significant difference between lesions (*p* = 0.035) regarding α-SMA immunoreactivity was observed (Fig. 1).

α-SMA expression was higher in larger RRC, with a significant positive and moderate correlation (*p* = 0.004). No statistical significance was observed for RC (Table 2).

**Figure 2:**
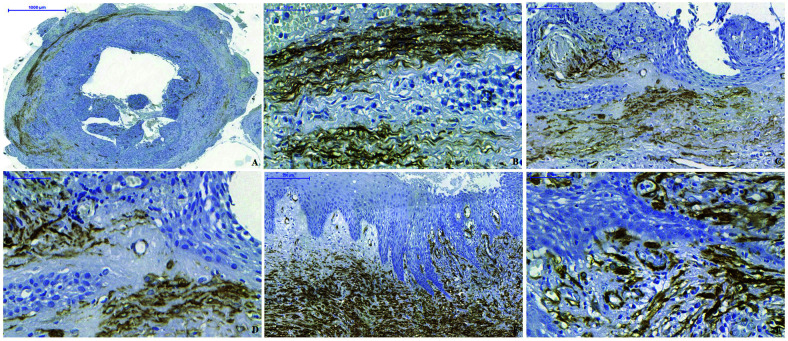
α-SMA immunostaining pattern. A) MF located deep in the connective tissue capsule in RC (Scale Bar: 1000 μm). B) MF arranged in bundles with the predominantly fusiform form, as well as, strong but focal α-SMA immunostaining in the connective tissue capsule containing intense inflammatory infiltrates in RC (Scale Bar: 50 μm). C) MF distributed diffusely and almost to the full extent of the connective tissue capsule in RRC (Scale Bar: 100 μm). D) MFs are predominantly ovoid and stellate in shape interspersed in the scarce inflammatory infiltrate in RRC (Scale Bar: 50 μm). E) Strong α-SMA immunostaining adjacent to the hyperplastic epithelium of RRC giving contractile force (Scale Bar: 100 μm). F) MFs are predominantly ovoid and fusiform in shape interspersed in the moderate inflammatory infiltrate in RRC (Scale Bar: 50 μm).

Regarding immunostaining intensity, RC showed strong focal α-SMA immunoexpression, located mainly in the deep portion of the fibrous connective tissue capsule, while RRC showed strong α-SMA immunoexpression but diffusely distributed throughout the fibrous connective tissue capsule (Fig. 2).

CD34 immunoexpression

Evaluation of immunoblotted lesions by the anti-CD34 antibody revealed immunoreactivity in endothelial cells. The angiogenic index obtained by MVC revealed a median of 60.1 (45.8-68.5) for RC and of 58.1 (41.8-75.1) for RRC. No statistically significant difference between lesions was observed (*p* = 0.988) (Table 1). A significant positive and moderate correlation (*p*=0.004) was found only between α-SMA and CD34 in RRC (Table 2).

**Figure 3:**
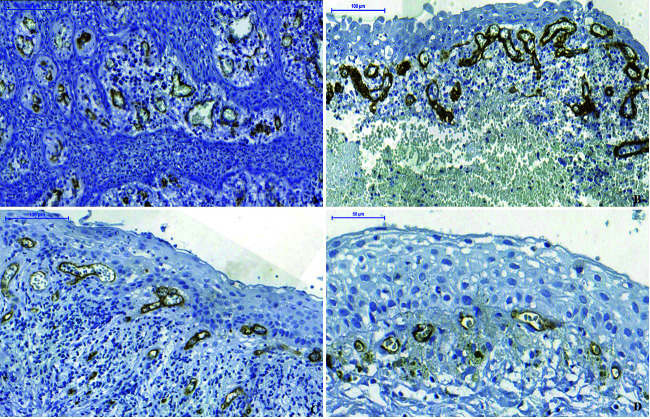
CD34 immunostaining pattern. A) Photomicrograph indicating large amounts of vessels immunostained with anti-CD34 antibody in hyperplastic epithelium of RC (Scale Bar: 100 μm). B) Numerous and larger caliber vessels immunostained strong for anti-CD34 antibody near the atrophic epithelial layer in RC with intense inflammatory infiltrates (Scale bar: 100 μm). C) Large vessels underlying the hiperplastic epithelium in RRC with intense inflammatory infiltrates (Scale bar: 100 μm). D) Small vessels underlying the atrophic epithelium in the scarce inflammatory infiltrates of RRC (Scale bar: 50 μm).

The highest concentration of CD34-immunstained vessels was located near the cystic epithelium and was not statistically significant related to inflammatory infiltrate intensity of epithelial thickness. Larger vessels were found in the subepithelial RCs and RRCs region (Fig. 3).

## Discussion

Periapical lesions represent a dynamic inflammatory reaction. The RRC originate from RC, which remained in the bone after inadequate or incomplete surgical procedures and continue their development (12). It is noteworthy that the growth and expansion mechanisms of these cysts are not completely understood.

RC and RRC present very similar histopathological characteristics although, the determining stimulus is present in RC and absent in RRC (12). Because these lesions are inflammatory in nature, all cases were classified according to inflammatory infiltrate intensity. The examined RC presented intense inflammatory infiltrates (70%), corroborating the studies carried out by Andrade *et al*. (13). In turn, most RRC presented mild (36.7%), moderate (36.7%) and intense (26.7%) inflammatory infiltrates, in line with the aforementioned studies (13). Thus, the fact that inflammatory infiltrate intensity was higher in RC is related to the intense metabolic activity observed in these lesions when compared to RRC, since the main antigenic stimuli are no longer present in the latter (13).

It is assumed that the hyperplastic epithelial lining is characteristic of active lesions. Thus, lesions exhibiting atrophic epithelia demonstrate a quiescence status (14). The present study revealed a higher frequency of lesions presenting hyperplastic epithelium (56.7%) compared to atrophic epithelium (43.3%) in RC, in accordance to previous studies (14). On the other hand, a higher number of lesions presenting atrophic epithelium (70%) when compared to hyperplastic epithelium (30%) was noted in RRC, similar to the aforementioned study (14).

MFs are characterized by actin microfilament bundles (stress fibers) containing smooth muscle α-actin, thus presenting high contractile capacity (15). MFs are transdifferentiated fibroblasts and the beta growth transforming factor (TGF-β) cytokine plays an important role in directly promoting their development by inducing α-SMA expression (10,16-17). This actin isoform predominates in vascular smooth muscle cells and its activation plays an important role in fibrotic response (18).

In this perspective, studies involving inflammatory odontogenic lesions reveal that MFs are related to cyst growth and expansion, modulating collagen deposition and angiogenesis mechanisms, as these cells are responsible for the synthesis of enzymes capable of degrading the extracellular matrix. Thereby controlling lesion growth and vascular support through angiogenesis (10,16). Unfortunately, few studies on the presence of MF in odontogenic cysts are available (2,5,10,18-23) and only four address inflammatory odontogenic lesions (16-17,24-25).

MF play an important role in inflammatory responses, as they secrete inflammatory mediators, growth factors, interstitial matrix molecules, chemokines and cytokines (5). Thus, the secretion of some factors such as TGF-ß1, monocyte chemotactic protein (MCP1) and platelet-derived growth factor (PDGF) have been implicated in the appearance of MF and, consequently in the growth of several cystic lesions (5,10).

In the present study, MF were present in almost all assessed lesions, with immunoreactivity confined to the cytoplasm and/or cell nucleus. A statistically significant difference between the presence of MF in the RCs and RRCs was observed, with higher concentrations in RRC. It is believed that the presence of MFs is essential in the physiological construction of scar processes, due to their ability to produce collagen, which may be associated to lesion growth and progression (18).

Growth factors produced by MF, including TGF-ß1 and tumor necrosis factor alpha (TNF-α), induce MMP secretion by these cellular elements, playing a role in extracellular matrix (ECM) remodeling, secreting metalloproteinase-2, 13 (MMPs-2, 13), plasminogen activator urokinase (uPA), as well as interacting with epithelial cells and connective tissue cells, and the activation of these enzymes can lead to the release and activation of sequestered cytokines and growth factors, which may favor angiogenesis and RC and RRC expansion (19). Additionally, MF contribute to bone resorption, particularly in RC (16). MF were present in significantly higher amounts in RRC, regardless of size. Larger RRC contained intense inflammatory infiltrates, thus justifying the role of inflammation in MMP production by MF and RRC expansion.

MFs were detected in almost all analyzed lesions in areas below the cystic epithelium and in the capsule depth, corroborating the assessment carried out by Sousa-Neto *et al*. (17), which detected MF in 100% of RC at an average of 4.66 cells per field, albeit at slightly lower amounts compared to the present study.

MF amounts were higher in RRC when compared to RC in the present study. MF are known to play a role in repair by promoting tissue remodeling through type I, III, IV, VIII collagen secretion, as well as glycoproteins such as fibronectin, tenascin, laminin, chondroitin sulfate, and matrix metalloproteinases MMP1, MMP2, and MMP3 (26). Understanding that repair represents one of the late-stage stages of inflammation, MF are believed to be higher in RRC because they are more chronic lesions than RC (4). Thus, inflammatory cells present in RRC are replaced in time by collagen, which would explain the higher amounts of MF detected in this type of lesion.

According to the results obtained herein, MF seem to play a double role in RRC, acting in the repair and containment of these lesions in cases of mild inflammatory infiltrate degrees, as well as in the growth of these periapicopathies in cases of intense inflammatory infiltrate degrees. Cystic growth containment is justified by MF location on RC peripheries and the greater dispersion of these cells by the connective tissue capsule of RRC. Consequently, as MF are more distributed in the RRC capsule, they will become closer to vessels and obtain access to more nutrients, allowing for collagen fiber deposition and RRC repair. It is noteworthy that cyst containment occurs during the quiescence phases and is more extensive in RRC. However, during cystic expansion phases, MF act on conjunctive remodeling through MMP released by MF, contributing to cystic expansion.

No statistically significant differences were observed concerning the interaction between inflammation and α-SMA, corroborating the study carried out by Sousa-Neto *et al*. (17) who indicated no or infrequent MF in inflammation areas, while reporting a greater amount of these cells in granulation tissue areas.

The expression of vascular markers may be associated with the inflammatory pattern and its progression (10). Vascular neoformation supports the inflammatory process, as new vessels transport oxygen and nutrients necessary for cellular demands to the inflammation site (3).

Few studies are available concerning the real role of vascularization in the development of periapicopathies. Connective tissue rich in newly formed blood vessels is essential for epithelial tissue maintenance, as it forms an ecosystem in which continuous communication occurs between participating cells. Capsule changes in these cystic lesions are dependent on MF, TGF-ß1 and PDGF released by epithelial cells, which are responsible for MF appearance, while low TGF-ß1 concentrations are strongly chemotactic for fibroblasts (10).

Based on this assumption, CD34 capsule expressions were analyzed. Angiogenesis measurements were performed through microvascular counting, as this is a simple, easy and effective method, according to Freitas *et al*. (27). According to Jordan *et al*. (28), poor endothelial cell immunostaining has been observed in some cases, as delayed fixation (more than 48 hours), inadequate dehydration, and excessive paraffin temperature (over 54 degrees) may impair the immunohistochemical technique and lead to immunostaining changes.

The CD34 marker analysis in RC and RRC indicated similar amounts of blood vessels in both lesions. No statistically significant differences were observed between CD34 and inflammatory infiltrates, corroborating the findings reported by Berar *et al*. (29). Disagreeing with from our results, which revealed a significant difference, this difference is probably due to the applied methodology (semi-quantitative) (30) and the different immunohistochemical marker applied (VEGF) (3).

A higher number of cells in CD34 immunopositive capsules was observed in RC, indicating this lesion presents higher metabolic and proliferative activity, therefore requiring a higher supply of oxygen and nutrients when compared to RRC (29). However, no significant difference was observed between CD34 expression and inflammatory infiltrate intensities in cystic lesions, although these immunostained cells are close to the inflammatory infiltrate, in agreement with other assessments (30), where authors indicate that some yet unknown factor may stimulate endothelial cell CD34 expression, regardless of inflammatory infiltrate intensity.

Potential correlations between the angiogenic index and the presence of MF in RC and RRC were also evaluated, only detected in RRC, corroborating another study (17), which found a positive and moderate correlation between these indices in RRC. In the case of odontogenic lesions, the presence of MF constitutes a source of angiogenic proteins, as well as extracellular matrix degradation proteinases, which together favor lesion growth (10,15).

It is also important to note that angiogenesis also assists cyst progression by stimulating the formation of new blood vessels, which increase oxygenation, allowing for the deposition of the fibrin-rich matrix essential for cell migration, greater accumulation of inflammatory cells and, consequently, the accumulation of more fluid in the cystic cavity (10). Based on this assumption, angiogenesis may act contributing to the growth and expansion of periapicopathies.

Based on the findings of the present study, this study concludes that immunohistochemical expression of α-SMA revealed greater immunostaining in RRC compared to RC. Considering inflammatory action, MF act favoring cystic expansion, while in the quiescence period of the lesion these cellular components act in tissue repair, as well as, α-SMA and CD34 immunoexpression indicate a moderate positive correlation between MF and the angiogenic index in RRC, allowing for the inference that these proteins may contribute to cystic growth containment.
